# Horizontal and vertical exoplanet thermal structure from a JWST spectroscopic eclipse map

**DOI:** 10.1038/s41550-025-02666-9

**Published:** 2025-10-28

**Authors:** Ryan C. Challener, Megan Weiner Mansfield, Patricio E. Cubillos, Anjali A. A. Piette, Louis-Philippe Coulombe, Hayley Beltz, Jasmina Blecic, Emily Rauscher, Jacob L. Bean, Björn Benneke, Eliza M.-R. Kempton, Joseph Harrington, Thaddeus D. Komacek, Vivien Parmentier, S. L. Casewell, Nicolas Iro, Luigi Mancini, Matthew C. Nixon, Michael Radica, Maria E. Steinrueck, Luis Welbanks, Natalie M. Batalha, Claudio Caceres, Ian J. M. Crossfield, Nicolas Crouzet, Jean-Michel Désert, Karan Molaverdikhani, Nikolay K. Nikolov, Enric Palle, Benjamin V. Rackham, Everett Schlawin, David K. Sing, Kevin B. Stevenson, Xianyu Tan, Jake D. Turner, Xi Zhang

**Affiliations:** 1 Department of Astronomy, Cornell University, Ithaca, NY USA; 2 Department of Astronomy, University of Michigan, Ann Arbor, MI USA; 3 The School of Earth and Space Exploration, Arizona State University, Tempe, AZ USA; 4 Department of Astronomy, University of Maryland, College Park, MD USA; 5 Space Research Institute, Austrian Academy of Sciences, Graz, Austria; 6 INAF - Turin Astrophysical Observatory, Pino Torinese, Italy; 7 Earth and Planets Laboratory, Carnegie Institution for Science, Washington DC, USA; 8 Department of Physics and Institute for Research on Exoplanets, Université de Montréal, Montreal, Quebec Canada; 9 Department of Physics, New York University Abu Dhabi, Abu Dhabi, United Arab Emirates; 10 Center for Astrophysics and Space Science (CASS), New York University Abu Dhabi, Abu Dhabi, United Arab Emirates; 11 Department of Astronomy and Astrophysics, University of Chicago, Chicago, IL USA; 12 Department of Earth, Planetary, and Space Sciences, University of California Los Angeles, Los Angeles, CA USA; 13 Department of Physics and Trottier Institute for Research on Exoplanets, Université de Montréal, Montreal, Quebec Canada; 14 Planetary Sciences Group, Department of Physics and Florida Space Institute, University of Central Florida, Orlando, FL USA; 15 Université Côte d’Azur, Observatoire de la Côte d’Azur, CNRS Laboratoire Lagrange, Nice, France; 16 School of Physics and Astronomy, University of Leicester, Leicester, UK; 17 Institute of Planetary Research, German Aerospace Center (DLR), Berlin, Germany; 18 Department of Physics, University of Rome “Tor Vergata”, Rome, Italy; 19 Max Planck Institute for Astronomy, Heidelberg, Germany; 20 Trottier Institute for Research on Exoplanets, University of Montréal, Montréal, Quebec Canada; 21 Department of Astronomy and Astrophysics, University of California Santa Cruz, Santa Cruz, CA USA; 22 Instituto de Astrofisica, Departamento de Ciencias Fisicas, Facultad de Ciencias Exactas, Universidad Andres Bello, Santiago, Chile; 23 Department of Physics and Astronomy, University of Kansas, Lawrence, KS USA; 24 Kapteyn Astronomical Institute, Rijksuniversiteit Groningen, Groningen, The Netherlands; 25 Anton Pannekoek Institute for Astronomy, University of Amsterdam, Amsterdam, The Netherlands; 26 Universitäts-Sternwarte, Ludwig-Maximilians-Universität München, Munich, Germany; 27 Exzellenzcluster Origins, Garching, Germany; 28 Space Telescope Science Institute, Baltimore, MD USA; 29 Instituto de Astrofísica de Canarias (IAC), Tenerife, Spain; 30 Department of Earth, Atmospheric and Planetary Sciences, Massachusetts Institute of Technology, Cambridge, MA USA; 31 Kavli Institute for Astrophysics and Space Research, Massachusetts Institute of Technology, Cambridge, MA USA; 32 Steward Observatory, University of Arizona, Tucson, AZ USA; 33 Department of Earth and Planetary Sciences, Johns Hopkins University, Baltimore, MD USA; 34 Department of Physics and Astronomy, Johns Hopkins University, Baltimore, MD USA; 35 Johns Hopkins APL, Laurel, MD USA; 36 Tsung-Dao Lee Institute, Shanghai Jiao Tong University, Shanghai, P. R. China; 37 Department of Earth and Planetary Sciences, University of California Santa Cruz, Santa Cruz, CA USA; 38 Present Address: School of Physics and Astronomy, University of Birmingham, Edgbaston, UK

**Keywords:** Exoplanets, Exoplanets, Atmospheric chemistry, Atmospheric dynamics

## Abstract

Highly irradiated giant exoplanets known ‘ultrahot Jupiters’ are anticipated to exhibit large variations of atmospheric temperature and chemistry as a function of longitude, latitude and altitude. Previous observations have hinted at these variations, but the existing data have been fundamentally restricted to probing hemisphere-integrated spectra, thereby providing only coarse information on atmospheric gradients. Here we present a spectroscopic eclipse map of an extrasolar planet, resolving the atmosphere in multiple dimensions simultaneously. We analyse a secondary eclipse of the ultrahot Jupiter WASP-18b observed with the Near Infrared Imager and Slitless Spectrograph instrument on the JWST. The mapping reveals weaker longitudinal temperature gradients than were predicted by theoretical models, indicating the importance of hydrogen dissociation and/or nightside clouds in shaping global thermal emission. In addition, we identify two thermally distinct regions of the planet’s atmosphere: a ‘hotspot’ surrounding the substellar point and a ‘ring’ near the dayside limbs. The hotspot region shows a strongly inverted thermal structure due to the presence of optical absorbers and a water abundance marginally lower than the hemispheric average, in accordance with theoretical predictions. The ring region shows colder temperatures and poorly constrained chemical abundances. Similar future analyses will reveal the three-dimensional thermal, chemical and dynamical properties of a broad range of exoplanet atmospheres.

## Abstract (web summary)

JWST data show that the exoplanet WASP-18b has thermally distinct regions of its dayside. The authors mapped the sizes of these regions, measured their temperatures and potentially identify water destruction in the hottest region.

## Main

As part of the James Webb Space Telescope (JWST) Early Release Science Program^1^, we observed a secondary eclipse of WASP-18b with the first order of the Near Infrared Imager and Slitless Spectrograph (NIRISS) Single-object Slitless Spectroscopy (SOSS) mode^2^ covering 0.85–2.85 μm. The dayside spectrum of the planet revealed an inverted vertical temperature profile, the presence of water in the atmosphere and evidence for short-wavelength absorbers such as H^−^, TiO or VO (ref. ^3^). A broadband eclipse map of the planet showed that the planet’s hottest hemisphere is aligned with the substellar point and there are steep temperature gradients from the substellar point to the limbs^3^, both indicators for atmospheric drag^4,5^.

Here we reanalyse the NIRISS data by applying the secondary eclipse mapping method at multiple wavelengths to infer the multidimensional temperature structure of WASP-18b’s atmosphere^6^. We used the Eigenspectra^7^ method to reanalyse the wavelength-resolved, systematics-corrected light curves presented in ref. ^3^. In brief, we fit a two-dimensional (2D) brightness map independently at each wavelength to the eclipse ingress, egress and out-of-eclipse phase variation^8^. As described in Methods, these fits were strongly preferred over simple sinusoid fits to the out-of-eclipse phase variation in more than half of the wavelength bins, with a Bayesian information criterion (BIC) difference of ΔBIC ≥ 10 in favour of the Eigenspectra model. Then, we stacked the individual wavelength maps together, identified spatial regions of the planet (groups) that are spectroscopically similar and extracted the spectra from each group. These spectra were then analysed with traditional one-dimensional (1D) characterization approaches to determine the vertical temperature structures and chemical compositions of each region. In this work, we used HyDRA^9–12^ and Pyrat Bay^13^ to make atmospheric inferences (that is, atmospheric retrieval). Thorough descriptions of Eigenspectra, the retrievals on Eigenspectra and a second eclipse mapping method (ThERESA) we used to cross-check our results can be found in Methods.

## Results

### Wavelength-dependent maps

**Fig. 1:**
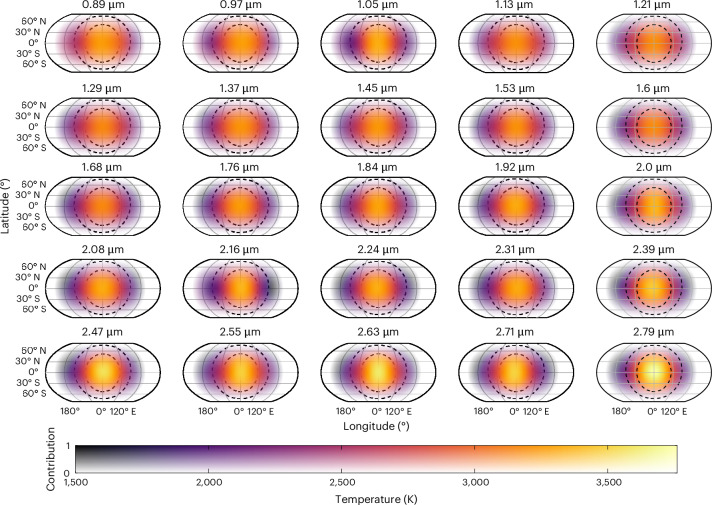
Two-dimensional maps from the Eigenspectra method for each of the 25 spectroscopic bins. The colours indicate the temperature and transparency indicates the relative contribution to the overall observed flux at the point of maximum visibility, based on the angle between a given point on the map and the line of sight to the observer. A maximum contribution of 1 indicates a latitude/longitude that is at the sub-observer point at some point during the observations. The dashed black curves delineate the three regions identified by the Eigenspectra mapping method. Evidence of multidimensional atmospheric structure can be seen in the varying hotspot temperature and shape with wavelength.

**Extended Data Fig. 1:**
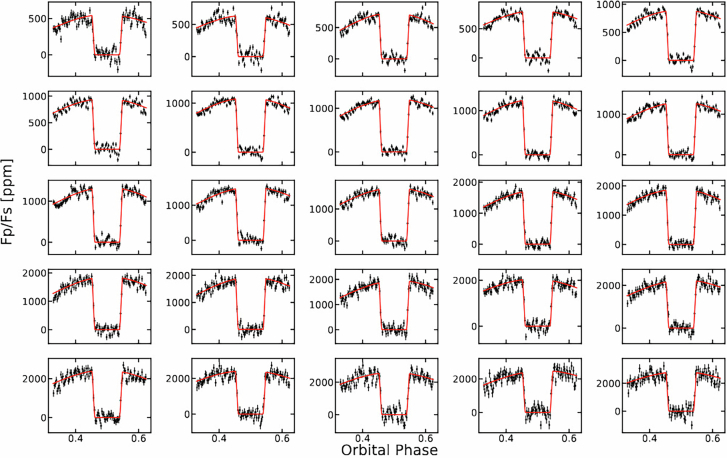
Light curve fits from the Eigenspectra method for each of the 25 spectroscopic bins. Black points with error bars indicate wavelength- and time-binned (weighted average), systematics-corrected data from Coulombe et al. (2023)^3^ with propagated uncertainties, and red lines show best fits from the Eigenspectra method. The light curves are shown in planet flux (*F*_*p*_) divided by stellar flux (*F*_*s*_). The models fit the data well and show differences in brightness with wavelength, showing evidence of multidimensional atmospheric structure in the data.

Because of the small size of eclipse mapping signals and the computational intensity of mapping fits, we performed eclipse mapping on 25 light curves binned down evenly in wavelength from the higher-resolution light curves fitted in ref. ^3^. We also tested a lower-resolution spectrum and found similar results (Methods). However, we note that, generally, the effect of wavelength resolution on spectroscopic eclipse mapping has not been studied in detail and should be examined in future work. Figure 1 shows the 2D brightness temperature maps for each of the 25 wavelengths, and Extended Data Fig. 1 shows the Eigenspectra fits to each of the 25 bins.

**Fig. 2:**
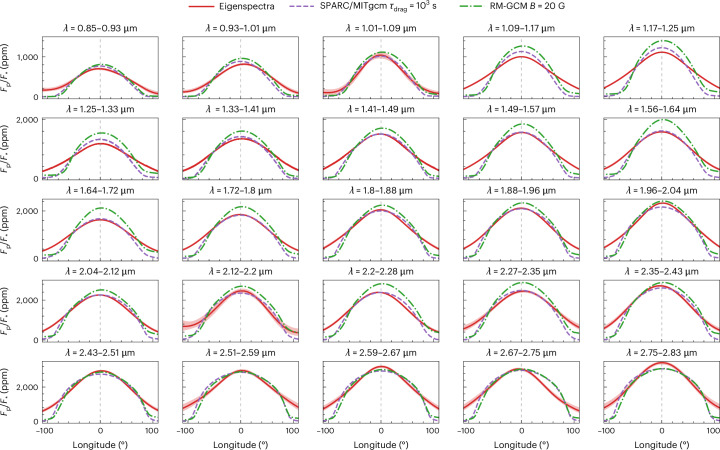
Retrieved longitudinal profiles at each wavelength range compared against GCMs. The Eigenspectra-retrieved profiles and GCMs including drag both show small hotspot offsets and sharp temperature gradients away from the substellar point at all wavelengths. The red lines and regions show the median retrieved longitudinal profiles and their 1*σ* confidence intervals, respectively, measured with Eigenspectra for the 25 spectral bins considered. The profiles are obtained by weighting the retrieved 2D maps by the squared cosine of the latitude. The profiles are compared with two GCMs from ref. ^3^ that matched the white-light map well—the SPARC/MITgcm (purple dashed line), which has uniform drag of timescale *τ*_drag_ = 10^3^ s, and the RM-GCM (green dash-dot line), which includes a kinematic magnetohydrodynamical drag model with an internal magnetic field of *B* ≈ 20 G. We note that the GCMs as shown here are processed to remove the ‘null space’ of components that are physically inaccessible to eclipse mapping (Methods and refs. ^39,40^). Vertical dashed lines indicate zero longitude.

Before stacking the single-wavelength maps, we constructed longitudinal brightness profiles by weighting the retrieved 2D maps from Eigenspectra by the squared cosine of the latitude. Figure 2 shows the longitudinal brightness profiles for Eigenspectra. Uncertainties on the longitudinal profiles from Eigenspectra are low (about 5–10%) because the data are well fit by only 2 or 3 non-uniform map components, depending on wavelength, limiting model flexibility to large-scale variations. Hotspot offsets from Eigenspectra ranged from −5° to 7°, with uncertainties of ~1° (Table 1). This trend of small to negligible hotspot offsets for all wavelengths examined is in agreement with the previous analysis of the full dayside observations^3^.

**Table 1:** The three group spectra extracted from Eigenspectra, using 25 bins evenly spaced in wavelength, in units of brightness temperature

Wavelength (μm)	Hotspot (K)	Ring (K)	Outer (K)	Hotspot offset (°)
0.85–0.93	3,135 ± 15	$2,80{9}_{-42}^{+40}$	$2,47{2}_{-144}^{+114}$	$1.{5}_{-3}^{+1}$
0.93–1.01	3,106 ± 14	$2,73{8}_{-35}^{+34}$	$2,34{5}_{-107}^{+89}$	−5.5 ± 2
1.01–1.09	$3,11{4}_{-37}^{+36}$	$2,60{1}_{-67}^{+62}$	$2,19{3}_{-182}^{+133}$	$-0.{5}_{-1}^{+2}$
1.09–1.17	3,046 ± 6	2,692 ± 11	$2,23{3}_{-26}^{+25}$	3.5 ± 1
1.17–1.25	3,032 ± 5	2,660 ± 9	$2,18{3}_{-22}^{+21}$	−4.5 ± 3
1.25–1.33	$2,98{9}_{-6}^{+5}$	2,611 ± 10	$2,13{3}_{-24}^{+23}$	$-2.{5}_{-0}^{+1}$
1.33–1.41	3,035 ± 6	2,628 ± 11	$2,12{6}_{-30}^{+29}$	$-1.{5}_{-0}^{+1}$
1.41–1.49	3,044 ± 6	2,608 ± 10	2,077 ± 22	$2.{5}_{-1}^{+0}$
1.49–1.56	3,005 ± 6	2,566 ± 11	$2,03{3}_{-23}^{+22}$	$3.{5}_{-4}^{+2}$
1.56–1.64	2,972 ± 8	2,520 ± 13	$1,99{4}_{-34}^{+32}$	−1.5 ± 1
1.64–1.72	2,943 ± 8	2,480 ± 13	$1,94{6}_{-29}^{+28}$	$1.{5}_{-2}^{+0}$
1.72–1.80	3,015 ± 8	2,514 ± 14	$1,94{4}_{-30}^{+29}$	$-0.{5}_{-1}^{+2}$
1.80–1.88	3,054 ± 10	2,542 ± 18	$1,95{6}_{-44}^{+42}$	$-3.{5}_{-2}^{+1}$
1.88–1.96	3,064 ± 8	2,521 ± 13	$1,92{2}_{-36}^{+34}$	−1.5 ± 1
1.96–2.04	3,110 ± 11	2,553 ± 8	$1,92{6}_{-34}^{+33}$	−1.5 ± 1
2.04–2.12	3,058 ± 12	2,493 ± 20	$1,86{3}_{-42}^{+41}$	$-5.{5}_{-1}^{+2}$
2.12–2.20	$3,05{7}_{-45}^{+44}$	$2,45{6}_{-94}^{+91}$	$2,07{9}_{-228}^{+195}$	$4.{5}_{-1}^{+2}$
2.20–2.27	3,038 ± 15	2,441 ± 24	$1,80{4}_{-47}^{+45}$	$-1.{5}_{-0}^{+1}$
2.27–2.35	3,047 ± 24	2,502 ± 42	$1,93{0}_{-113}^{+104}$	$-0.{5}_{-1}^{+0}$
2.35–2.43	3,151 ± 21	$2,51{7}_{-35}^{+34}$	$1,84{5}_{-86}^{+80}$	2.5 ± 1
2.43–2.51	3,232 ± 20	2,559 ± 32	$1,87{2}_{-65}^{+62}$	$-1.{5}_{-3}^{+1}$
2.51–2.59	3,187 ± 32	$2,58{1}_{-56}^{+55}$	$1,96{7}_{-138}^{+127}$	$1.{5}_{-0}^{+1}$
2.59–2.67	3,271 ± 29	$2,59{5}_{-49}^{+48}$	$1,90{5}_{-115}^{+107}$	$-0.{5}_{-2}^{+4}$
2.67–2.75	$3,17{0}_{-34}^{+33}$	$251{2}_{-56}^{+55}$	$1,85{3}_{-131}^{+121}$	7.5 ± 1
2.75–2.83	3,362 ± 40	$2,63{1}_{-65}^{+64}$	$1,92{4}_{-143}^{+132}$	$2.{5}_{-1}^{+2}$

We also list the hotspot offset and error bar at each wavelength. Note that all offsets are half-integer values and there are some wavelengths where the 1*σ* error bar appears to be 0. This is because of the longitude grid cell spacing (1°), which naturally allows for only half-integer values and integer error bars.

Figure 2 also compares the retrieved longitudinal profiles with predictions from two general circulation models (GCMs) previously compared with the full secondary eclipse spectrum^3^: the SPARC/MITgcm^14^, which includes a uniform drag, and the RM-GCM^5^, which uses a kinematic magnetohydrodynamic drag. Both GCMs were computed assuming solar atmospheric metallicity and carbon-to-oxygen ratio. We note that, at nearly all wavelengths, the RM-GCM is brighter than the SPARC/MITgcm, which is probably due to different radiative transfer methods resulting in different thermal structures between the two GCMs. The observed lack of substantial hotspot offsets agrees with the GCMs, as expected based on predictions that ultrahot Jupiters will experience increased magnetic atmospheric drag, slowing the planet’s equatorial jet^5,15^. More complex treatments of magnetohydrodynamic additionally predict oscillating hotspot offsets due to nonlinear Reynolds stresses^16,17^, which could be confirmed with follow-up observations at different epochs.

Spectroscopic eclipse mapping observations are in theory able to probe the temperature structure and hotspot offset over a range of pressures because the photospheric pressure changes with wavelength. For hot Jupiters such as WASP-18b, GCMs generally predict increasing hotspot offsets at deeper pressures^5,18^. We searched for trends in the hotspot offset as a function of retrieved pressure but found no clear trends. This may be due to the difficulty of seeing trends in hotspot offset for WASP-18b in particular, which as one of the hottest known ultrahot Jupiters is predicted to have smaller hotspot offsets than slightly cooler planets throughout much of its atmosphere^19^. Future work to apply spectroscopic eclipse mapping to cooler planets, which are expected to have more variation in hotspot position, may reveal stronger trends.

Notably, the GCMs also predict a steeper decrease in flux away from the substellar point than the Eigenspectra maps at most wavelengths, leading to cooler predicted temperatures near the limb at ±90° longitude. GCMs with weaker drag cannot account for this difference, as they all show larger hotspot offsets than what is seen in the Eigenspectra longitudinal profiles. The warmer-than-predicted limbs may be due to the influence of hydrogen dissociation and recombination, which increases day–night heat transport^7,15^. We tested models including H_2_ dissociation and a uniform drag and found no substantial warming near the limbs. However, future models combining H_2_ dissociation and a non-uniform drag may result in more limb warming. Alternatively, the warm limbs could be indicative of nightside clouds, which would warm the atmosphere and potentially change the substellar-point-to-limb temperature gradient^20,21^, but are not included in the GCMs shown here.

### Horizontal and vertical map

**Fig. 3:**
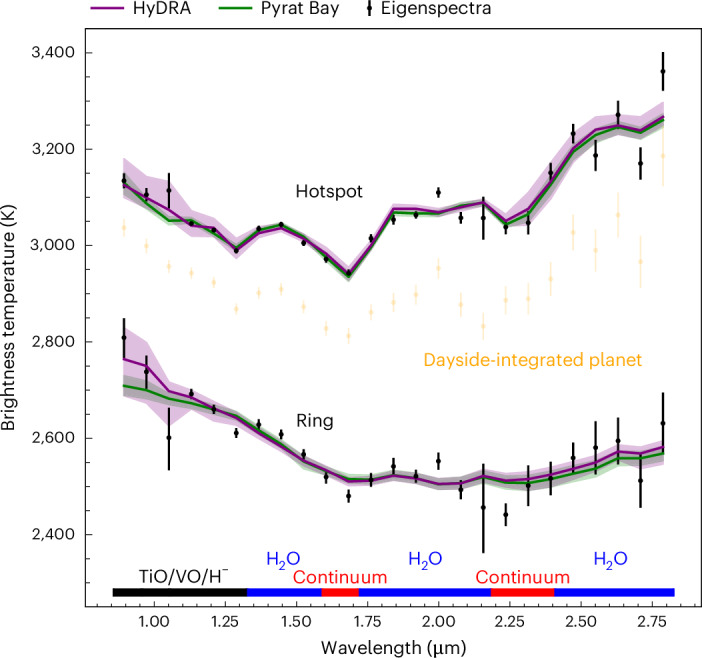
Hotspot and ring-group spectra from Eigenspectra bracket the full dayside-integrated spectrum. Black points with error bars (standard deviation; Methods) show the Eigenspectra emission spectra from the hotspot and ring groups, and yellow points show the hemispherically averaged homogeneous dayside emission spectrum^3^, binned in wavelength to match the group spectra. The spectra have been converted to brightness temperature by assuming blackbody emission for the planet in each bin and a PHOENIX emission model for the star. The HyDRA (purple) and Pyrat Bay (green) regions show 95.45% (2*σ*) credible regions from 1D atmospheric retrievals on each spectrum. Labels along the bottom show the wavelength ranges at which different atmospheric constituents create features in the spectrum. An average of the flux from the hotspot and ring regions produces a spectrum matching the dayside average (Extended Data Fig. 2). See Methods for further discussion of the ring spectrum and the mismatch with associated retrievals.

**Extended Data Fig. 2:**
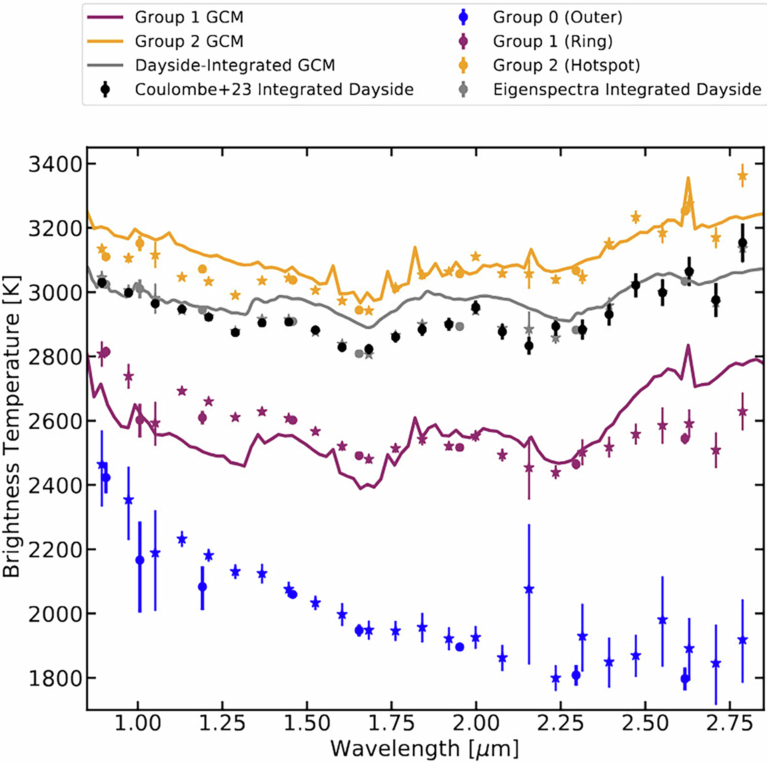
Comparison of group spectra resulting from Eigenspectra and the full hemisphere-integrated dayside spectra on different resolution wavelength binning schemes. Black points show the full secondary eclipse spectrum from ref. ^3^ binned to the 25 wavelength bins discussed in the main text. Yellow, purple, and grey spectra show the output from Eigenspectra for the hotspot group, ring group, and the full hemisphere-integrated dayside spectrum. In all cases, stars show the higher resolution scheme with 25 wavelength bins discussed in the main text, while dots indicate the 8-wavelength bin scheme used in the methods for comparison to ThERESA. The hemisphere-integrated dayside spectrum from Eigenspectra closely matches the spectrum from ref. ^3^, with all points within 1.2*σ* of the original spectrum, indicating that the fit is performing well. Additionally, there is little difference in the shapes of the spectra at higher vs. lower resolution. Lines show the dayside-integrated spectrum and grouped spectra from SPARC/MITgcm output. The predicted group spectra from the GCM provide a relatively good match to the Eigenspectra. For completeness, blue points show the output from Eigenspectra for the outer group, which was not analyzed fully due to its small contribution to the overall signal during the observation. Error bars are the standard deviation (see Methods).

We then applied the full Eigenspectra method to identify spatial regions with similarly shaped spectra. As shown in Fig. 1, the Eigenspectra mapping method identified three regions of the map with distinct spectral shapes, which are roughly concentric circles centred on the substellar point. This demonstrates the multidimensional information in these data, and the need for a multidimensional approach to interpreting them: if a uniform planet (that is, one with even no phase variation out of eclipse) was a reasonable assumption within our data precision, Eigenspectra would find only one distinct region in the planet. We note that these three groups are a discrete approximation of a planet that probably has continuously varying properties, which we discuss further in Methods. We refer to these three groups as the ‘hotspot’, ‘ring’ and ‘outer’ groups, in order of their angular distance from the substellar point. The outer group, shown in Extended Data Fig. 2, had a signal-to-noise ratio about 2–12× lower than the other groups because it contains regions of the planet observed only very briefly near the beginning or end of the observation as part of the nightside rotated into view (Table 1). Therefore, we limit our analysis to the hotspot and ring groups. Figure 3 shows the emission spectra from the hotspot and ring groups, along with 1D best-fitting models to those spectra. These spectra, as expected, bracket the hemispherically averaged dayside spectrum from ref. ^3^, with the hotspot spectrum ~150 K hotter and the ring spectrum ~400 K colder. Indeed, an average of the flux emitted from these regions, when appropriately accounting for viewing geometry and relative area, closely matches the dayside average emission spectrum (Extended Data Fig. 2).

## Discussion

**Fig. 4:**
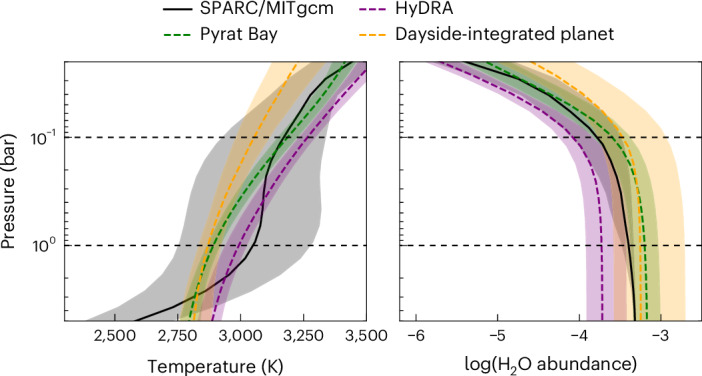
The Eigenspectra hotspot group shows a slightly hotter temperature and slightly lower water abundance than the full dayside-integrated result. The left plot shows temperature–pressure profiles and the right plot shows retrieved H_2_O abundances. In both plots, purple and green lines show retrievals on the Eigenspectra hotspot spectrum using HyDRA and Pyrat Bay, respectively, and yellow lines show the retrieval on the full dayside spectrum from ref. ^3^, with shading indicating 68.3% credible region (1*σ*) uncertainties. The black lines show average profiles in the group region from a SPARC/MITgcm of solar atmosphere metallicity and carbon-to-oxygen ratio, and the black-shaded regions show the full range of per-point GCM profiles in the region of the hotspot group. The black dashed lines indicate the approximate range of pressures that our observation probes. Note that the full planet retrieval was also performed with HyDRA, and so is more similar in retrieval set-up to the HyDRA hotspot retrieval than that of Pyrat Bay. For reference, the equilibrium temperature of WASP-18b is ~2,400 K (ref. ^72^).

Atmospheric inference of the hotspot group shows a thermal structure and composition that is consistent with the same approach applied to the full dayside spectrum, and similar to expectations from GCMs (Fig. 4). The hotspot thermal profile is marginally hotter than the dayside average but shows a similar thermal inversion (increasing temperature with decreasing pressure) at the planet’s near-infrared photosphere, which is likewise predicted by GCMs. The retrieved H_2_O abundances are consistent with the GCM but marginally lower than the full dayside retrieval, probably due to factors such as increased thermal dissociation of H_2_O in the hotter hotspot group. While the retrievals do not tightly constrain any other individual chemical abundances, we find evidence for optical opacity sources (a combination of H^−^, TiO and VO), probably drivers of the thermal inversion, at 5.1*σ*. Evidence for optical opacity is also seen in the spatially averaged dayside spectrum (a weighted average of the hotspot, ring and outer spectra), although with a slightly lower detection significance of 4.6*σ*. Such similarities are expected, as the hotspot region is both bright and directly visible throughout the observation, and therefore dominates the planet’s dayside emission. However, changes in chemistry and vertical temperature gradient away from the hotspot may weaken the detection of optical species in the averaged dayside spectrum. In addition, while the hotspot thermal structure and water abundance are not significantly different from those of the full dayside, the marginal shifts observed are consistent with the theoretical expectation that the hottest region of the planet should show the most water dissociation^22^. This illustrates the utility of the Eigenspectra method to isolate parts of the dayside with stronger spectral features, with the potential to strengthen chemical detection significances.

The ring-group spectrum is qualitatively similar to GCM predictions, which show water emission features but at a brightness temperature ~500 K colder than the hotspot region (Extended Data Fig. 2). However, we also found that the retrieved temperature–pressure profile and chemical abundances for the ring group depended sensitively on how the models account for geometric effects such as the different average line of sight through the atmosphere in the ring group compared with a standard full dayside secondary eclipse. We discuss the ring-group results further in Methods.

While previous observations with Spitzer, Hubble and JWST led to key advancements in our understanding of hot exoplanets, they were fundamentally limited to hemisphere-integrated spectra^21,23–26^ or single-wavelength photometric maps^3,8,27,28^. The spectroscopic eclipse mapping presented here is an observational analysis that resolves multidimensional information at multiple wavelengths simultaneously. Our findings are consistent with predictions of water dissociation in the hottest part of the atmosphere^22^ and indicate the importance of hydrogen dissociation^15^ and/or nightside clouds^20,21^ in shaping substellar-to-limb temperature gradients. Moving forward, the large wavelength coverage and high precision of JWST will enable similar multidimensional mapping for a large sample of exoplanet atmospheres, allowing the study of horizontal and vertical thermal and chemical gradients across a population of giant exoplanets. Through comparison with theoretical predictions from GCMs, these maps will place crucial constraints on atmospheric dynamics and chemical transitions.

## Methods

We applied two complementary spectroscopic eclipse mapping methods to the data: Eigenspectra^7^ and ThERESA^29^. We used two methods because, as described below, they interpret the data in distinct ways, giving us a way to check which conclusions derived from the eclipse maps are robust to differences in mapping methods. While Eigenspectra fits the spectroscopic data well, ThERESA struggled to match the emission features in the data, as ThERESA simultaneously fits a three-dimensional (3D) model to all the spectroscopic light curves, giving the model less flexibility than fitting each spectroscopic bin individually. Therefore, we chose to highlight only the Eigenspectra method in the main text, but we include ThERESA here as an independent check to verify some of the main results from Eigenspectra. Below, we describe some potential paths for future research to investigate how to improve multiwavelength eclipse mapping. Both methods start with the wavelength-resolved, systematics-corrected light curves presented in ref. ^3^.

### Mapping with Eigenspectra

The Eigenspectra method^7^ splits 3D eclipse mapping into two stages. In the first stage, 2D brightness temperature maps are constructed for each wavelength bin following the eigenmapping method^8^. First, we derive an orthogonal basis set of light curves from spherical-harmonic light curves using principal component analysis, where each light curve has a corresponding 2D map component. Then, we perform a 2D fit at each wavelength using a subset of this basis set of light curves. The fitting begins with a small number of components, and the number of components is increased until the BIC indicates that the addition of more components is not preferred. For the fits presented in the main text, the 2D mapping preferred 4 or 5 free parameters at each wavelength, except for the fit at 1.05 μm, which preferred 7 free parameters. To perform multiwavelength mapping, Eigenspectra then extracts spectra from a grid of points in latitude and longitude across the visible area of the planet and uses a *k*-means clustering algorithm to identify regions of the planet with similarly shaped spectra. This grouping is repeated in a Markov-chain Monte Carlo (MCMC) framework to estimate uncertainties in the resulting grouped spectra.

The number of distinct groups is chosen by starting with one group and increasing the number one by one until the largest number of groups for which individual pixels are sorted into the same group across 75% of the MCMC map iterations is identified. This ensures that the number of groups is limited by the ability of the data to precisely sort latitude/longitude points into the best-fit group. We note that the 75% cut-off was chosen arbitrarily based on visually inspecting the results for different numbers of groups. Future work to perform Eigenspectra mapping on a larger sample of planets should further investigate whether this cut-off holds across different datasets. Supplementary Fig. 1 shows the mean group maps and histograms of the assigned group for randomly chosen points on the map for the 25-bin analysis described in the main text. The data showed a clear separation between groups when using two or three groups, but became mixed when using four groups.

We note that the grouping performed by Eigenspectra creates discrete spectra, but these discrete spectra probably represent a true planet with continuous properties, such as a smooth temperature gradient. The *k*-means clustering allows us to set the number of groups by the precision of the data, such that a more precise dataset would be able to identify more spectra, because the change in properties required to distinguish two spectra with smaller error bars is correspondingly smaller. In the limit of infinitely precise data, each latitude/longitude point would be identified as a distinct group with distinct properties. However, this discrete representation allows us to determine how much the properties change across the visible area of the planet in a way that is regulated by the signal to noise of the real observational data.

For each identified group, the Eigenspectra method then creates a representative spectrum by taking an area- and visibility-weighted mean of the spectrum of each point included in the group, and scaling it by an area weighting to represent it on the same scale as a regular secondary eclipse spectrum, which covers a full visible hemisphere of a planet. The primary outputs of this mapping method are therefore a handful of spectra representing emission from different regions on the planet, which are run through atmospheric inference (retrieval) codes to measure molecular abundances and thermal structures. By virtue of the area and visibility weighting, the eigenspectra are mathematically defined such that they should produce a hemisphere-integrated brightness equivalent to that of the full eclipse spectrum. Extended Data Fig. 2 shows that the hemisphere-integrated brightness indeed matches the wavelength-binned eclipse spectrum from ref. ^3^.

The error bars on the grouped spectra are calculated by taking the standard deviation of each point included in a group across all MCMC realizations of the planet map. The MCMC runs used 100 walkers, 7,000 steps and a burn-in of 700 steps. Convergence was evaluated by ensuring that the chain was at least 50 times as long as the autocorrelation timescale (see the emcee^30^ documentation at https://emcee.readthedocs.io/en/stable/tutorials/autocorr/). After calculating the errors in this way, we found that the hemisphere-integrated brightness had slightly smaller error bars than those from the original dayside eclipse spectrum from ref. ^3^. We tested running retrievals in the same format as the fiducial retrievals but with the error bars scaled up by a factor to match the original dayside eclipse spectrum, and we found that this change did not impact the retrieval results.

The method described above closely follows the method for mapping with Eigenspectra described in ref. ^7^, with two key improvements. First, for the 2D mapping with the eigencurves method, we restricted allowed planet maps to those which produce positive fluxes at all observed latitudes and longitudes, as a realistic planet must have positive thermal emission. Second, the area weighting was applied to the resulting mean spectra for each group to allow atmospheric retrieval with standard secondary eclipse retrieval codes. We computed per-point spectra on a grid with a resolution of 1° in both latitude and longitude. We also tested grids with a resolution of 3° and 9° and found that the positions of the groupings did not depend on the grid resolution.

The analysis described in the main text used 25 wavelength bins evenly spaced between 0.85 μm and 2.83 μm, with a width of 0.079 μm. We achieve reduced *χ*^2^ values of 1.02–1.39 for the single-wavelength eigenmapping fits, with between 4 and 7 free parameters per fit and 2,719 data points, and an overall ${\chi }_{\nu }^{2}=1.19$ for the full multiwavelength eigenmapping fit. The best fits at each wavelength were obtained with a small number of eigenmapping components, restricting the resulting maps to the large-scale patterns characteristic of low-order spherical harmonics. The ${\chi }_{\nu }^{2}$ values at each wavelength are slightly above the expected value of 1 for a fit with correctly estimated error bars. These elevated values are likely because the spectroscopic light curves were corrected for systematics at a higher resolution by ref. ^3^ and then later binned down for use in this work. We recommend that future work investigate removing systematics at the same wavelength resolution at which eclipse mapping fits are performed, and/or performing simultaneous systematics and eclipse mapping fits.

In addition to the 25-wavelength-bin fit described in the main text, we tested whether the results depended on the wavelength resolution by running a lower-resolution Eigenspectra fit. The lower-resolution fit had eight wavelength bins, with their central wavelengths and widths optimally chosen to capture spectral features seen in the original secondary eclipse spectrum (Table 1). The light curve fits for the 8 wavelength bins had reduced *χ*^2^ values between 1.22 and 2.26, with between 4 and 6 free parameters per fit and 2,719 data points. The larger reduced *χ*^2^ values are probably due to the greater amount of binning applied to the original data. We found that the temperature maps had the same shape as for the 25-bin fit, and the Eigenspectra method still identified 3 distinct spectral groups in nested rings. In addition, atmospheric retrievals on the 8-bin hotspot and ring groups showed consistent results with the 25-bin retrievals. We ultimately used the 25-bin spectrum for the main results because of the greater spectral resolution, but we used the 8-bin spectrum for comparison with the more computationally intensive ThERESA method, which could not be run on a greater number of wavelength bins within a reasonable timeframe.

To provide a quantitative analysis of how much of our mapping information comes from the phase-curve variation versus the eclipse itself, we compared our fit against one where we allow for only phase-curve variation, represented by a double sine function, and assume a standard box-shaped eclipse with no additional perturbations to the shape of ingress or egress. We used a double sine function to match the fit to the out-of-eclipse variation performed by ref. ^3^. The double sine fit had four free parameters, comparable to the four to seven free parameters in the Eigenspectra fits. This model is unphysical, as a planet with phase-curve variation necessarily has spatial brightness gradients and so should also induce a signal during ingress and egress, but with this approach we are artificially requiring the eclipse shape to match that of a planet with uniform brightness. A comparison between these two fits then reveals how much signal is contributed solely from the eclipse. We computed the BIC for both models and found a ΔBIC between −3 and 713 depending on the wavelength, with a positive number indicating a preference for the Eigenspectra fit. For 17 of the 25 wavelength bins, the ΔBIC was 10, indicating a strong preference for the Eigenspectra fit over the sinusoid fit. However, at some wavelengths, the improvement is marginal. This lack of strong preference for the eclipse mapping fit over a sinusoidal fit is due to several factors, including: (1) WASP-18b rotates substantially during our eclipse observation, creating considerable phase-curve variation that is present in a large part of the dataset relative to ingress and egress, and (2) WASP-18b’s low impact parameter reduces the strength of signatures of latitudinal temperature variation. However, the Eigenspectra analysis still provides multidimensional information that is not obtained from a simple sinusoidal phase variation fit—namely, the Eigenspectra fit reveals the radial extent of the hotspot so that its composition can be inferred separately from the surrounding dayside. Other planets will probably be even better targets for 2D and 3D characterization with JWST^31^.

The component of eclipse mapping that can be uniquely inferred through secondary eclipses and not out-of-eclipse phase-curve variation is latitudinal structure. Although the Eigenspectra maps show a lack of any latitudinal offset, this does not reflect an inability to constrain latitudinal information. To test the ability of the Eigenspectra fits to constrain latitudinal information, we follow methods similar to ref. ^32^ and artificially inject a latitudinal offset into the observations. Supplementary Figs. 2 and 3 show the light curves resulting from the minimum and maximum latitudinal offsets that produce fits with *χ*^2^ ≤ 10 higher than the best fit in each wavelength bin. We found that this requirement results in a median constraint on the latitudinal offset of −29° to 61°. This comparison demonstrates that the Eigenspectra method would be able to detect latitudinal structure outside this range if such structure existed.

Table 1 lists the grouped 25-bin spectra resulting from the Eigenspectra analysis used in the main text, and Supplementary Table 1 lists the 8-bin spectra used for comparison with ThERESA. The hotspot, ring and outer groups had a mean signal to noise of 483, 226 and 90, respectively. As described in the main text, although Eigenspectra identified three groups, we chose to fully analyse only two, the hotspot and ring. We chose not to apply atmospheric retrievals to the outer group because it had a signal-to-noise factor of ~2.5–12× smaller than the other spectra and a much smaller contribution to the secondary eclipse signal. While this may not appear to be a sizable difference in signal to noise, it probably indicates that the shape of the outer group is being driven by the fitting method rather than the data. As described above, the best 2D fit at each wavelength has a small number of eigenmapping components, and therefore will show only large-scale patterns characteristic of low-order spherical harmonics. Therefore, we suspect the shape of the outer group is primarily driven by the requirement of a smoothly varying map consistent with the much higher signal-to-noise hotspot and ring regions.

### Mapping with ThERESA

Similar to Eigenspectra, ThERESA splits 3D eclipse mapping into two stages: 2D mapping and 3D mapping. First, it constructs 2D star-normalized flux maps of the planet at each wavelength bin in the observation, using the eigenmapping method^8^. This methodology is identical to 2D mapping with Eigenspectra (Supplementary Fig. 4), although with ThERESA we use only eight spectroscopic bins (the aforementioned lower-resolution fit; Supplementary Table 1) to reduce the model complexity (see below). To ensure physically plausible maps, we enforce a positive-flux constraint at the longitudes that are visible during the observation (−134.7–151.8°). To convert these flux maps into brightness temperature maps, we first compute a grid of planet brightness temperature versus star-normalized planet flux, assuming that the planet emits as a blackbody and using a PHOENIX^33^ model for the stellar spectrum. Then we interpolate this grid to the fluxes in our observed maps to determine the brightness temperatures of the the maps. Because these maps cover a relatively small wavelength range and our wavelength bins are chosen to probe small pressure ranges, converting these flux maps to brightness temperature maps is a reasonable choice for the 3D mapping (described below).

For the 3D mapping, ThERESA parameterizes the vertical placement of each of the 2D brightness temperature maps. We test both a simple parameterization, where each 2D temperature map is placed at a single pressure level, and a more complex parameterization where the depth of the temperature map has a sinusoidal dependence on latitude and longitude, and the phase of the longitudinal sinusoid is allowed to vary. Effectively, this sinusoidal model allows the photosphere to shift vertically with changing instellation and the resultant impact on temperature. The 3D model also includes an internal temperature parameter that sets the temperature of the bottom of the atmosphere at all latitudes and longitudes. We linearly interpolate, in log(pressures), along each column of the atmosphere between the 2D temperature maps and the internal temperature to create a 3D temperature grid. The atmosphere is assumed to be isothermal above the highest-altitude 2D temperature map.

We then apply solar-abundance thermochemical equilibrium to each cell of the 3D temperature grid to calculate the atmosphere’s chemical composition. For computational speed, we precompute a grid of chemical abundances versus temperature and pressure using GGChem^34^, and then interpolate to the temperatures in the model atmosphere. Based in part on 1D atmospheric characterization of WASP-18b^3^, we include H_2_O, CO, CO_2_, TiO, VO and H^−^ in the atmosphere. We then calculate an emission spectrum from each column of the atmosphere using TauREx^35^ and integrate over the visible part of the atmosphere at each observation time, including the effects of planetary rotation, the angle between the sub-observer point and each grid cell, the area of each grid cell, and the occultation by the star. We use ExoTransmit^36^ molecular opacities and compute the model at the opacity native resolution (*R* ≈ 1,000), which is then binned to the data resolution. The model has 100 pressure layers evenly placed in log space between 0.0001 bar and 100 bar.

The resulting spectroscopic light curves are then compared against the data. This process (3D temperature grid parameterization, composition calculation, emission spectra calculation and spatial integration) is repeated behind an MCMC routine to explore the parameter space. For MCMC, we use the MC3 package^37^, which implements differential evolution Markov chains that can efficiently sample high-dimensional (>50) parameter spaces using a low number of chains^38^. We use 7 chains and run a total of ~1.4 million total iterations. For comparison with the Eigenspectra mapping, we achieve autocorrelation lengths of 21–50 for each parameter in the model. We calculate contribution functions for each spectroscopic bin and apply a penalty to the model goodness of fit if the vertical positions of the 2D brightness temperature maps are inconsistent with the contribution functions^29^. This penalty is a confidence-region-like calculation where, if the vertical position of a given 2D map falls within the pressures where 68.3% (1*σ*) of planetary emission at that wavelength originates, then there is effectively no penalty, but at substantially higher or lower pressures the penalty effectively causes the model to be rejected.

The full 3D temperature map is shown in Supplementary Fig. 5. Broadly, the 3D temperature structures agree with Eigenspectra, with a thermal inversion near the substellar point that transitions to roughly isothermal near the limbs. We achieve a reduced *χ*^2^ of 1.56 over all the spectroscopic light curves (33 model parameters, 21,752 data points), slightly worse than the Eigenspectra (Fig. 1) or the 2D ThERESA (Supplementary Fig. 4) fits. When considering all wavelengths together, the model residuals are well behaved and distributed Gaussian-like around zero. However, we note that ThERESA systematically overestimates or underestimates the light curves at certain wavelengths, and if the 3D model is post-processed into emission spectra (assuming thermochemical equilibrium and solar atomic abundances) from the planetary regions defined by Eigenspectra, the ThERESA spectra struggle to match the H_2_O features in the data. Models that create these emission features are within the parameter space we explored, but these models are rejected because they require increasing the temperature of the upper atmosphere, which leads to an overestimation of the total planetary emission, and such models also violate the contribution–function–consistency criterion. Because of this discrepancy with the observed spectra, we opt to only report the Eigenspectra results in the main text, which do not experience similar difficulties in fitting the spectroscopic data.

This mismatch motivates several avenues for additional work to understand 3D atmospheric retrieval with JWST data. First, ThERESA assumes that the planet’s upper atmosphere is isothermal, an assumption that worked well for synthetic data based on GCMs^29^ but may depress molecular emission features necessary to fit these data. Adjustments to the thermal profile parameterization that allow for flexibility in upper atmospheric temperature gradients would probably give the model the capability to match stronger molecular features. Second, ThERESA aims to place 2D brightness temperature maps at the pressures corresponding to contribution function maxima, to prevent non-physical scenarios where the 2D maps are placed at extremely high or low pressures. In reality, these 2D brightness temperatures come from a range of pressures, so the corresponding emission over that range, not just the emission at the peak of the contribution function, should be consistent with the 2D maps. Modifying the contribution function consistency check in this way would probably also reduce the model’s chances of creating an extended isothermal upper atmosphere. Finally, for simplicity, ThERESA assumes thermochemical equilibrium at solar atomic abundances. Expanding this framework to fit for bulk metallicity and C/O ratio, for example, could give the model some of the additional flexibility it needs to fit the data.

### Eclipse mapping null space

Some finer-resolution spatial flux patterns are inaccessible to eclipse mapping analyses, as they create zero signal during the observation^39,40^. These patterns, collectively referred to as the eclipse mapping null space, need to be removed from GCMs before comparing them against measured eclipse maps, as the measured maps will never place constraints on the null-space patterns. The GCMs presented in Fig. 2 have been processed to remove the null space by representing the GCMs as high-degree spherical-harmonic maps, using principal component analysis to identify null components of the map, and removing those null components^40^.

### Retrievals on Eigenspectra

#### HyDRA

HyDRA^9–12^ is an atmospheric-retrieval framework that combines a parametric forward atmospheric model with a nested sampling Bayesian parameter estimation algorithm, PYMULTINEST^41–43^. The inputs to the forward model include six parameters for the temperature–pressure profile, and the deep-atmosphere abundances of each of the chemical species considered. In particular, we use the temperature–pressure profile parameterization of ref. ^44^, which has been used extensively for atmospheric retrievals of exoplanet atmospheres, including ultrahot Jupiters such as WASP-18b^3,10^. The model also includes the abundances of chemical species that have opacity in the 0.8–2.8 μm range and are expected in H_2_-rich atmospheres^10,45^: collision-induced absorption due to H_2_–H_2_ and H_2_–He (ref. ^46^), H_2_O (ref. ^47^), CO (ref. ^47^), CO_2_ (ref. ^47^), HCN (ref. ^48^), OH (ref. ^47^), TiO (ref. ^49^), VO (ref. ^50^), FeH (ref. ^51^), Na (ref. ^52^), K (ref. ^52^) and H^−^ (refs. ^53,54^). For each opacity source, line-by-line absorption cross-sections^55^ are calculated using data from the references listed. The opacity from H^−^ free–free and bound–free transitions is calculated using the methods of refs. ^53,54^, respectively. We additionally include the effects of thermal dissociation for H_2_O, TiO, VO and H^−^. The depletion in the abundances of these species is calculated as a function of pressure and temperature, using a parametric method^22^. For all other species, the abundances are assumed to be constant with depth. In some of the retrievals, we additionally test the effects of adding a ‘dilution’ parameter (an area covering fraction), which multiplies the overall emission spectrum by a constant factor between 0 and 1 (ref. ^56^).

The forward model computes the thermal emission spectrum of the atmosphere given the input parameters described above. The pressure range considered is 10^−5^–10^3^ bar. The spectrum is calculated at a resolving power of *R* ≈ 15,000, and is convolved to the resolution of the instrument before being binned to the data resolution. The binned model is compared with the data to calculate the likelihood of the model instance. We use 2,000 live points in the nested sampling parameter estimation algorithm. HyDRA ultimately outputs the posterior probability distributions for each model parameter, from which we calculate the median and 1*σ* contours for the retrieved spectrum, temperature profile and chemical abundance profiles. We additionally perform Bayesian model comparisons to determine the evidence for one model (for example, including a particular molecule) over another (for example, which excludes that molecule). To do this, we compare the Bayesian evidence from the retrievals using each model, which we convert to a ‘sigma’ confidence value using the methods of ref. ^57^.

#### Pyrat Bay modelling framework

Pyrat Bay is an open-source framework that enables atmospheric modelling, spectral synthesis and Bayesian retrievals of exoplanet observations^13^. The atmospheric model consists of 1D parametric profiles of the temperature, volume mixing ratios (VMRs) and altitude as a function of pressure (hydrostatic equilibrium). For this analysis, we considered a pressure array extending from 10^−9^ bar to 100 bar and a wavelength array from 0.8 μm to 3.0 μm sampled at a resolving power of *R* = 15,000. The temperature profile follows the parametric prescription of ref. ^44^. Our framework computes abundances in thermochemical equilibrium via a Gibbs free-energy optimization code that combines the flexibility and performance of previous chemical frameworks^58,59^. This chemical code produces VMR profiles consistent with the pressure, temperature and elemental composition of the atmosphere at each layer. The chemical network includes 45 neutral and ionic species that are the main carriers of H, He, C, N, O, Na, Si, S, K, Ti, V and Fe. We adopted three free parameters to vary the elemental composition at each iteration: a carbon-abundance scaling factor ([C/H], relative to solar values), an oxygen scaling factor ([O/H]) and a third ‘catch-all’ parameter that scales the abundance of all other metals ([M/H]). The altitude of each layer is calculated assuming hydrostatic equilibrium. Finally, we also considered a free parameter for dilution^56^, which accounts for spatial inhomogeneities of the planetary flux.

For a given set of atmospheric parameters, Pyrat Bay computes the emission spectrum considering opacities from the Na and K resonant lines^60^; H, H_2_ and He Rayleigh scattering^61^; H_2_–H_2_ and H_2_–He collision-induced absorption^62–67^; H^−^ free–free and bound–free opacity^54^; and molecular line lists for CO, VO, H_2_O and TiO (refs. ^49,50,68,69^). To process the large molecular line-list opacity files, we applied the REPACK package^70^ to extract the dominant line transitions, which we then sampled over a temperature, pressure and wavelength grid for interpolation during retrieval runs. The Bayesian sampling in Pyrat Bay is managed with the MC3 package^37^, in this case using the MULTINEST nested sampling algorithm^41,42^ with 1,500 live points.

#### Hotspot group retrievals

Supplementary Figs. 6 and 7 show the results from retrievals on the hotspot group. Both retrievals of the hotspot group find a strong thermal inversion around the ~1 bar pressure level, where the temperature increases from 2,900 K to 3,300 K. Above this level, most molecules start to thermally dissociate, depleting the upper layers of the main optical/near-infrared absorbers (H_2_O, TiO and H^−^). The retrieved spectra are dominated by a series of H_2_O emission bands at wavelength *λ* > 1.25 μm and by optical opacity (for example, H^−^, TiO and/or VO) at *λ* < 1.5 *μ*m. The Pyrat Bay retrieval shows a well-constrained posterior with subsolar elemental abundances ([M/H] = −0.22 ± 0.16) and a subsolar C/O ratio (C/O = 0.22 ± 0.15); these elemental compositions lead to a water abundance of ${\log }\ {{n}_{{{\rm{H}}}_{2}{\rm{O}}}}=-3.20\pm 0.17$ at the photosphere. Similarly, the HyDRA retrieval shows a well-constrained water abundance of ${\log }\ {{n}_{{{\rm{H}}}_{2}{\rm{O}}}}=-3.{7}_{-0.2}^{+0.3}$, although it is unable to precisely constrain the abundances of any other species. These results generally agree with the full dayside atmospheric constraints^3^, which is expected as the bright and directly visible hotspot dominates thermal emission throughout the observation.

#### Ring-group retrievals

Supplementary Figs. 8 and 9 show a summary of retrieved constraints for the ring group. We note that we saw the same results for the ring group when using 8 and 25 wavelength bins. The nominal atmospheric retrievals of the ring group, as well as the ThERESA fit, result in physical properties in stark contrast to the hotspot group, although this depends strongly on the model assumptions, as described below. The nominal models result in non-thermally inverted temperature profiles with brightness temperatures of ~2,500–2,700 K, probed mainly at pressures of 1–10 bar by the observations. This decrease in temperature from ~3,000–3,200 K of the hotspot (Fig. 3) is roughly consistent with the GCMs with atmospheric drag, although the GCM temperatures in the ring region vary considerably with latitude/longitude and show thermal inversions.

Perhaps the most puzzling outcome of the ring-group retrieval is the atmospheric composition. With Pyrat Bay, we found that the abundance posterior distribution was constrained to the C/O > 1 region, leading to extremely low H_2_O abundances (VMR < 10^−6^), such that there were no visible H_2_O absorption bands in the model. Similarly, the nominal HyDRA retrievals on the ring group found very low H_2_O abundances (also VMR < 10^−6^), while we would expect VMR ≈ 10^−3.3^ (Supplementary Fig. 8). In both sets of retrievals, the ring spectrum was mainly dominated by absorption due to H_2_–H_2_ and H_2_–He collision-induced absorption. This represents a drop of over two orders of magnitude in H_2_O abundance from the hotspot to the ring group. Such a steep gradient in dayside composition seems physically unlikely, especially as H_2_O is expected to be more abundant in the cooler ring region compared with the hotspot, where thermal dissociation depletes the H_2_O abundance. In addition, the ring-group spectrum appears by eye to show slight H_2_O emission features at the same wavelengths where emission features are seen in the hotspot and full dayside spectra (for example, slight peaks at ~1.4 μm and ~1.9 μm; Supplementary Fig. 9). It is, therefore, possible that H_2_O absorption is shaping the ring-group spectrum, but is incorrectly identified in the retrievals. Finally, the lack of any detected opacity aside from H_2_–H_2_ and H_2_–He collision-induced absorption in the ring group throws into question the validity of the retrieved temperature–pressure profile, as the retrievals would not be sensitive to a wide range of pressures without any species that can change the atmospheric opacity over the wavelengths we investigated.

We suspect that there are physical or geometric effects that the 1D models are not able to capture, hence preventing the retrievals from providing a sound physical interpretation. We found that the standard model was strongly preferred over a simple blackbody (>14*σ*). The fact that the spectrum shows significant deviations from a blackbody indicates that the results of the standard retrieval are not due to an inability to detect atmospheric features; the data show a clear preference for a model with features over a perfect blackbody.

As a test, we ran additional retrievals fitting the standard model with the addition of a dilution parameter. Supplementary Fig. 9 shows the spectrum for the HyDRA code.

One possible explanation for these results is that what may be a sharper boundary in spectral features between the hotspot and ring groups is smeared out by the 2D eigencurves fitting. Eigencurves fitting is based on maps constructed from relatively low-order spherical harmonics, so it is fundamentally limited to producing maps with relatively smooth gradients^7,8^. If the true planet showed a rapid change in spectral features at a sharp boundary, the eigencurve mapping may smear out this sharp boundary, producing a mix of spectral features in the resulting group spectra that might confuse standard retrievals. However, we note that the grouped spectra are very similar in shape and amplitude to spectra derived from similar regions of a GCM, perhaps indicating that the eigencurve fitting does not have an oversized impact on the resulting spectra. The specific extent to which eigencurve fitting impacts the spectra can be investigated in the future by applying the Eigenspectra mapping method to GCM outputs where the ground-truth map is known.

We also explored whether the slant viewing angle between the observer and the flux from the ring-group biases the retrievals. For this, we modified the 1D emission models to, instead of integrating the planet intensity over the entire dayside hemisphere, integrating only over a region delimited by $\cos (\psi )\in (0.6,0.2)$, where *ψ* is the angle between the line of sight and the intensity vector over the dayside hemisphere. This is the region where the ring-group flux originates. While we confirmed that the slant viewing angle has a wavelength-dependent impact on the emission spectra, we found no significant changes in the retrieved temperatures or abundances between this approach and the nominal retrieval. However, we have not ruled out the possibility that some other effect due to the non-standard geometry may be impacting the retrievals. Temperature variations within the ring-group region could also affect the retrieval results. Indeed, the dilution parameter is designed to account for thermal inhomogeneities due to a hotspot region^56^, and could be compensating for variations within the relatively large ring-group region, although imperfectly.

We exclude the chemistry results from the ring group from the main text due to our suspicions that the retrievals may be impacted by some combination of the factors listed above. While a more detailed investigation of these possibilities is outside the scope of this work, future research should investigate this further to improve upon spectroscopic eclipse mapping methods. Applying the Eigenspectra method to GCM outputs would allow an investigation of the effects listed above and whether improving upon any of them can increase the fidelity of the retrievals.

## Supplementary information

Supplementary Information Supplementary Table 1 and Figs. 1–9.

## Data Availability

The data used in this work are publicly available in the Mikulski Archive for Space Telescopes (https://archive.stsci.edu/). The data that was used to create all of the figures in this paper are freely available on Zenodo^71^.

## Code availability

The open-source spectroscopic eclipse mapping pipelines used throughout this work are available at https://github.com/meganmansfield/eigenspectra (Eigenspectra) and https://github.com/rychallener/ThERESA (ThERESA). The Pyrat Bay atmospheric-retrieval package is available at https://github.com/pcubillos/pyratbay.
