# Supplementary material for: Horizontal and vertical exoplanet thermal structure from a JWST spectroscopic eclipse map
Source: Nat Astron. 2025 Oct 28;9(12):1821–32. doi: 10.1038/s41550-025-02666-9 (PMC12708353; doi:10.1038/s41550-025-02666-9)
Supplement: Supplementary file 1 — Supplementary Table 1 and Figs. 1–9. [file 41550_2025_2666_MOESM1_ESM.pdf]

nature astronomy

Supplementary information https://doi.org/10.1038/s41550-025-02666-9

Horizontal and vertical exoplanet thermal structure from a JWST spectroscopic eclipse map

In the format provided by the authors and unedited

Wavelength [μm]	Hotspot [K]	Ring [K]	Outer [K]	Hotspot Offset [°]
$0.86 - 0.96$	3110 ± 8	2814 ± 16	2423^{+49}_{-45}	1.5^{+1}_{-2}
$0.96 - 1.06$	3152 ± 24	2602^{+54}_{-51}	2166^{+163}_{-120}	2.5^{+1}_{-1}
$1.06 - 1.33$	3072 ± 9	2609 ± 21	2083^{+72}_{-64}	1.5^{+0}_{-2}
$1.33 - 1.59$	3037 ± 3	2601 ± 5	2059 ± 10	-1.5^{+1}_{-0}
$1.59 - 1.72$	2944 ± 6	2492 ± 10	1948 ± 19	-0.5^{+0}_{-1}
$1.72 - 2.18$	3057 ± 4	2517 ± 6	1896 ± 9	-1.5^{+0}_{-1}
$2.18 - 2.41$	3067 ± 9	2464 ± 15	1808^{+33}_{-32}	1.5^{+1}_{-2}
$2.41 - 2.83$	3252 ± 11	2544 ± 17	1797^{+36}_{-35}	-0.5^{+2}_{-1}

Supplementary Table 1 | Same as Table 1, but for the 8 optimally-chosen wavelength bins.

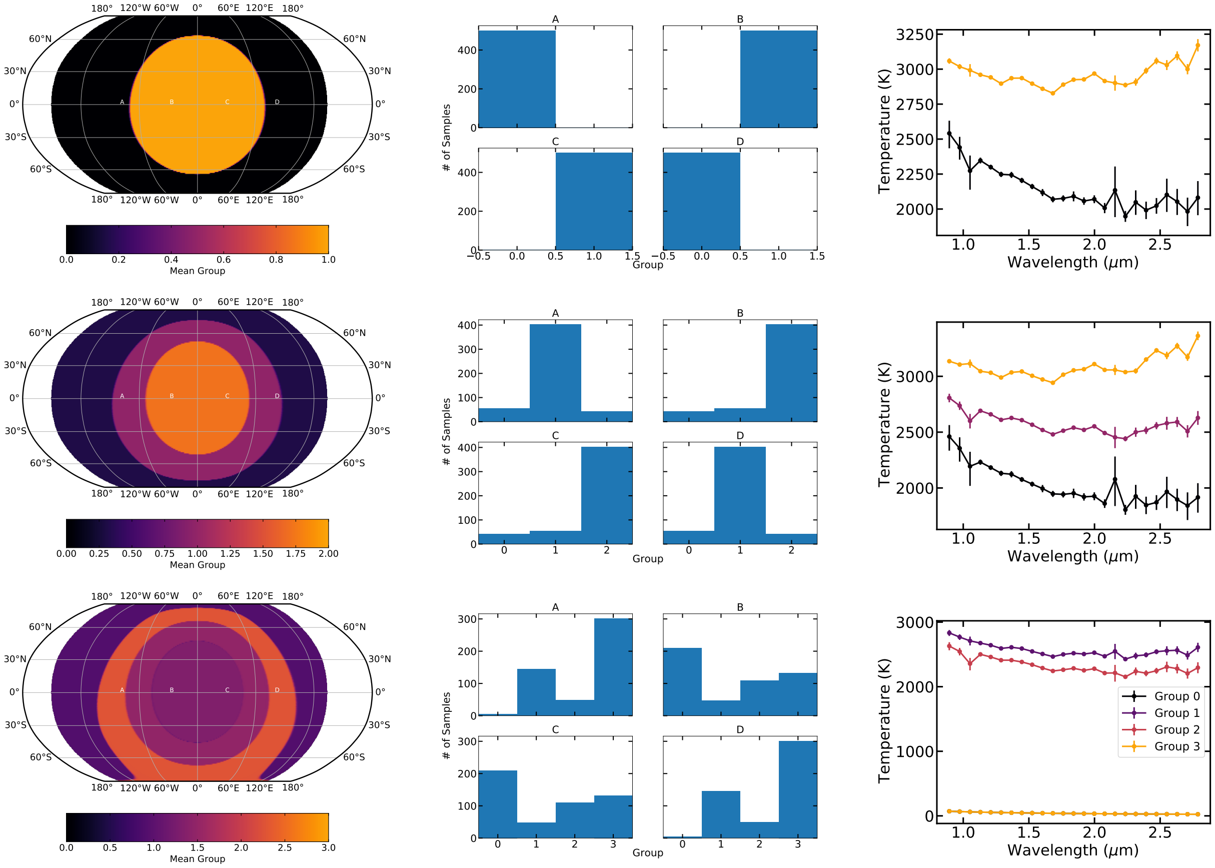

Supplementary Figure 1 | Mean group (left), histograms of grouping across several MCMC samples (middle), and resulting eigenspectra (right) for `Eigenspectra` mapping fits using 2 (top), 3 (middle), and 4 (bottom) groups. For each set of plots, histograms are labelled by letters which are overplotted on the map in the latitude/longitude position from which they are drawn (positions were chosen both near and far from group edges). Groups 2 and 1 here correspond to the hotspot and ring groups discussed in the text. For 3 groups, the map shows a clear division between groups and all of the points show $\geq 75\%$ of points assigned to a single group. For 2 groups, there is a similarly clear division, and notably the hotspot and outer groups have quite similar spectra to the corresponding groups in the 3-group case. For 4 groups, the mean group map does not show a clear division of groups, and the histograms show that the same point is sorted into different groups depending on the posterior draw. Additionally, the resulting spectra are not distinct (the group 0 spectrum is identical to the group 4 spectrum, which is why it is not visible on the plot). Therefore, we used 3 groups for this fit. Error bars on group spectra are the standard deviation (see Methods).

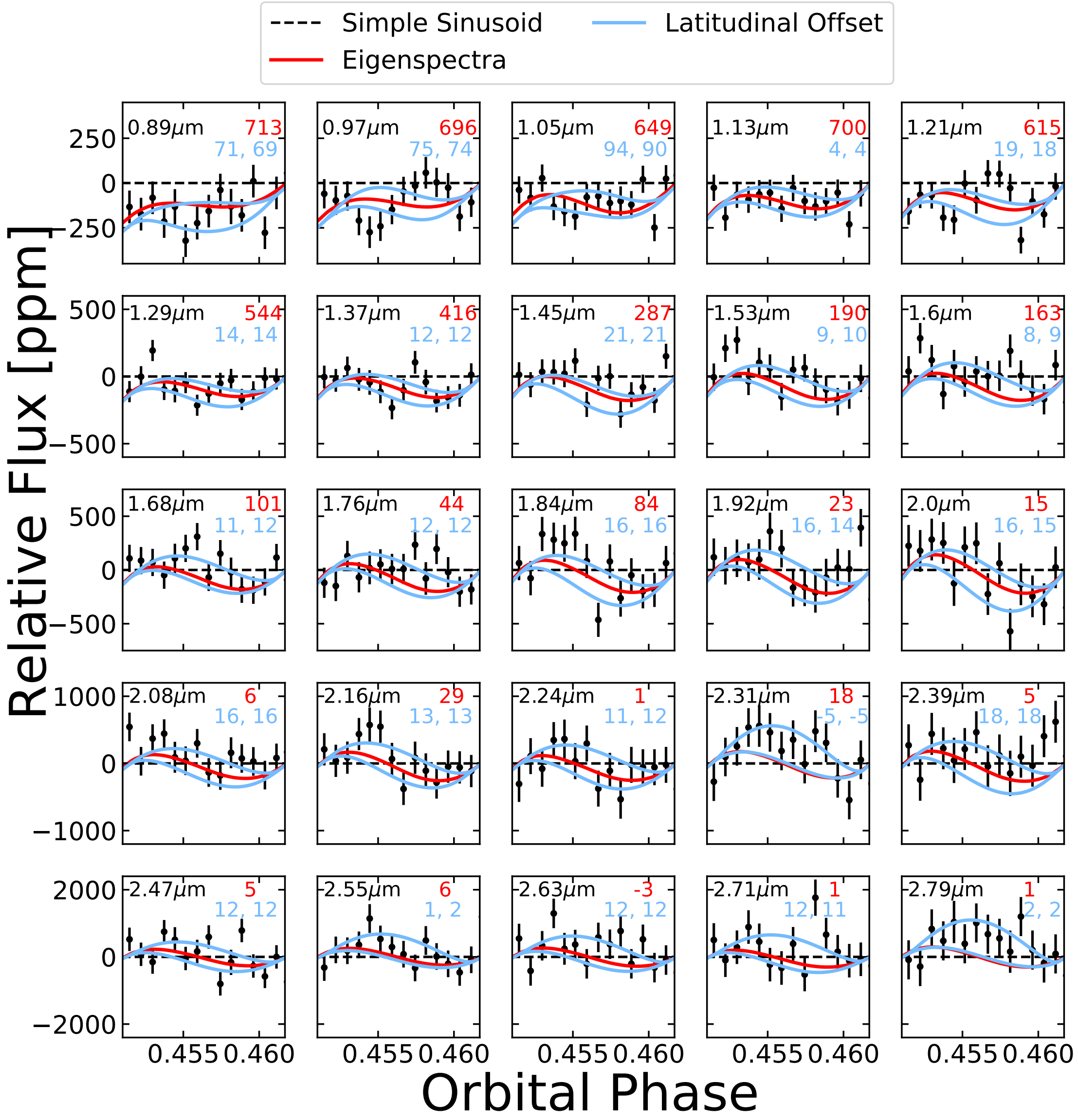

Supplementary Figure 2 | Zoom-in on ingress for each of the 25 wavelength bins in the `Eigenspectra` fit. Panels show relative flux (weighted averages with propagated uncertainties from ref. [3]) compared to the sinusoid fit described in the Methods (black lines), the real `Eigenspectra` fit (red), and the range of latitudinal offsets allowed for a fit with $\Delta\chi^2 \leq 10$ (blue lines). Black points with error bars show the difference between the binned data and the sinusoid fit. Numbers in the upper right corners show the ΔBIC for the fit to both ingress and egress between the sinusoid and `Eigenspectra` (red) and between the minimum and maximum latitudinal offset and `Eigenspectra` (blue). Positive numbers indicate a lower BIC was achieved by the `Eigenspectra` fit. For the majority of wavelengths, the `Eigenspectra` fit achieves a ΔBIC ≥ 10, indicating a strong preference for `Eigenspectra`. The `Eigenspectra` fit is not as strongly preferred at longer wavelengths where larger error bars allow for a wider range of suitable fits.

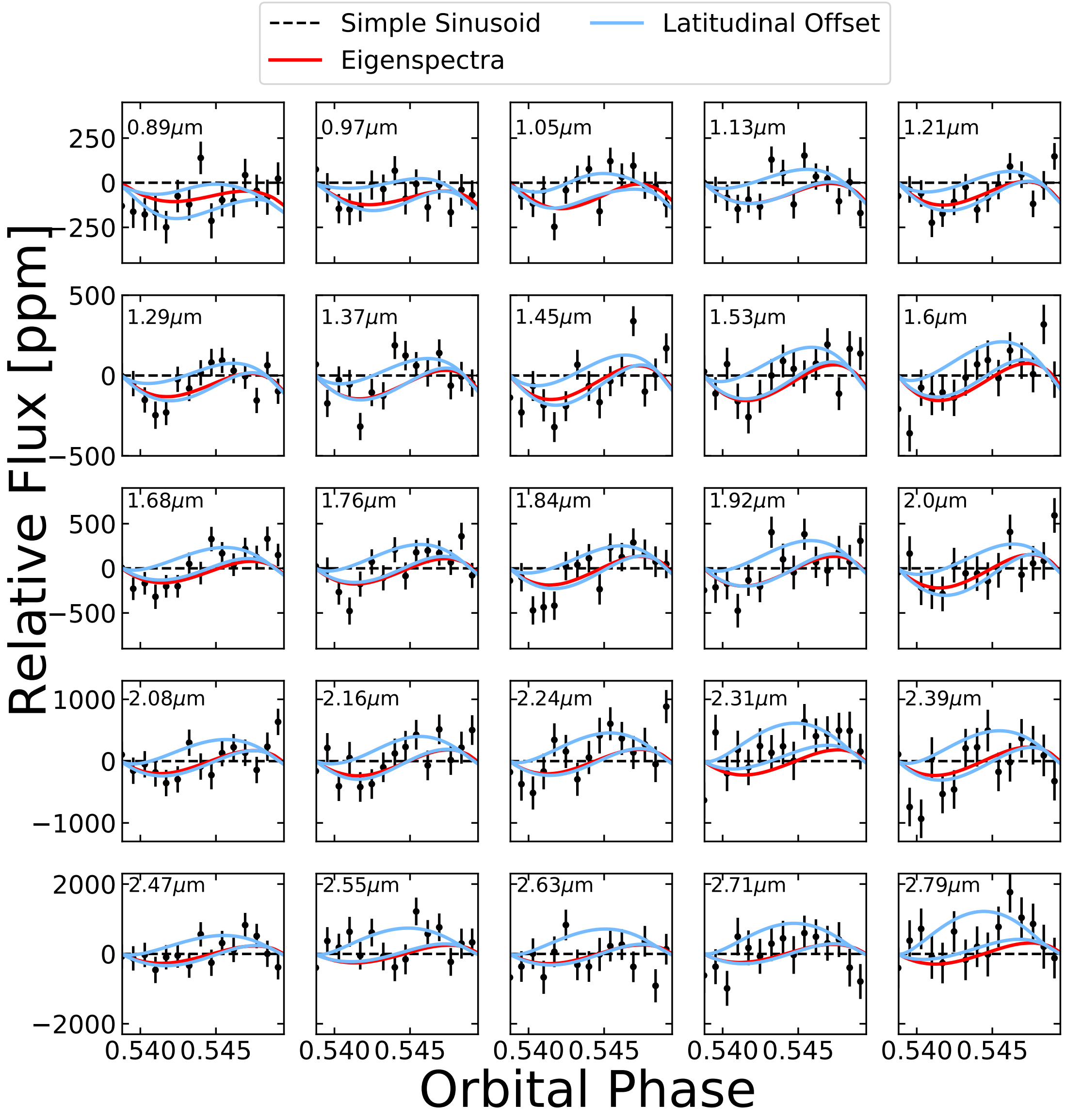

Supplementary Figure 3 | Same as Supplementary Figure 2, but showing the egress at each wavelength.

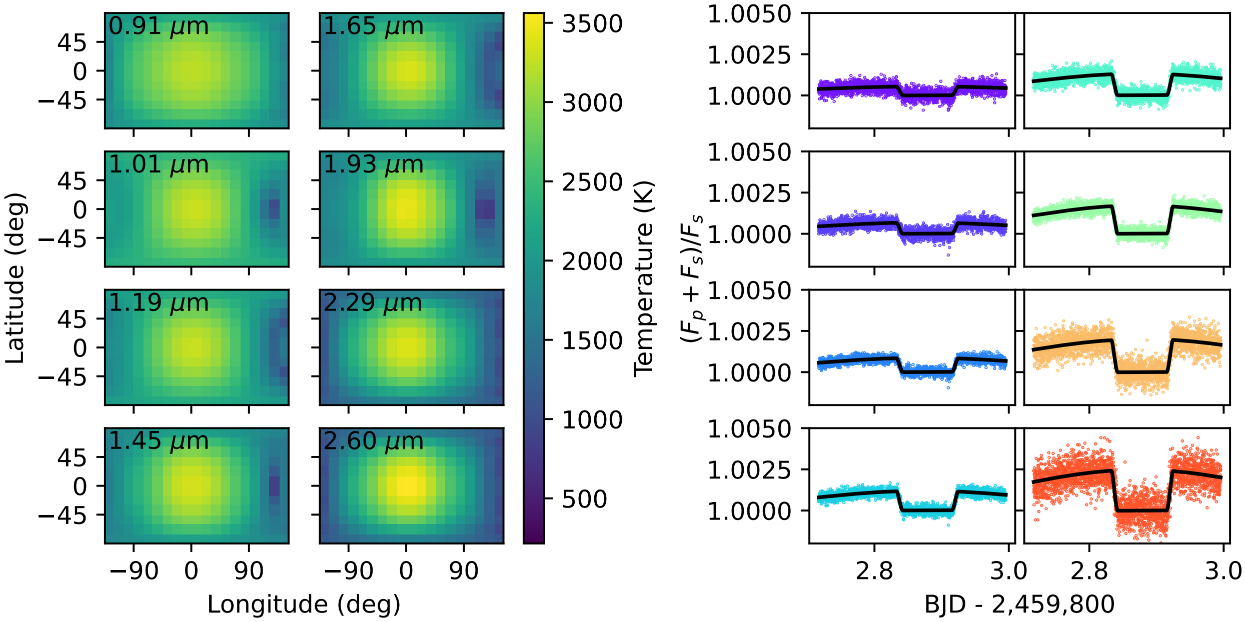

Supplementary Figure 4 | 2D brightness temperature maps from `ThERESA`, at each of the 8-wavelength bins, and the corresponding light-curve fits. The 2D mapping method is the same as used in `Eigenspectra`, and the resulting maps are consistent with those in Figure 1.

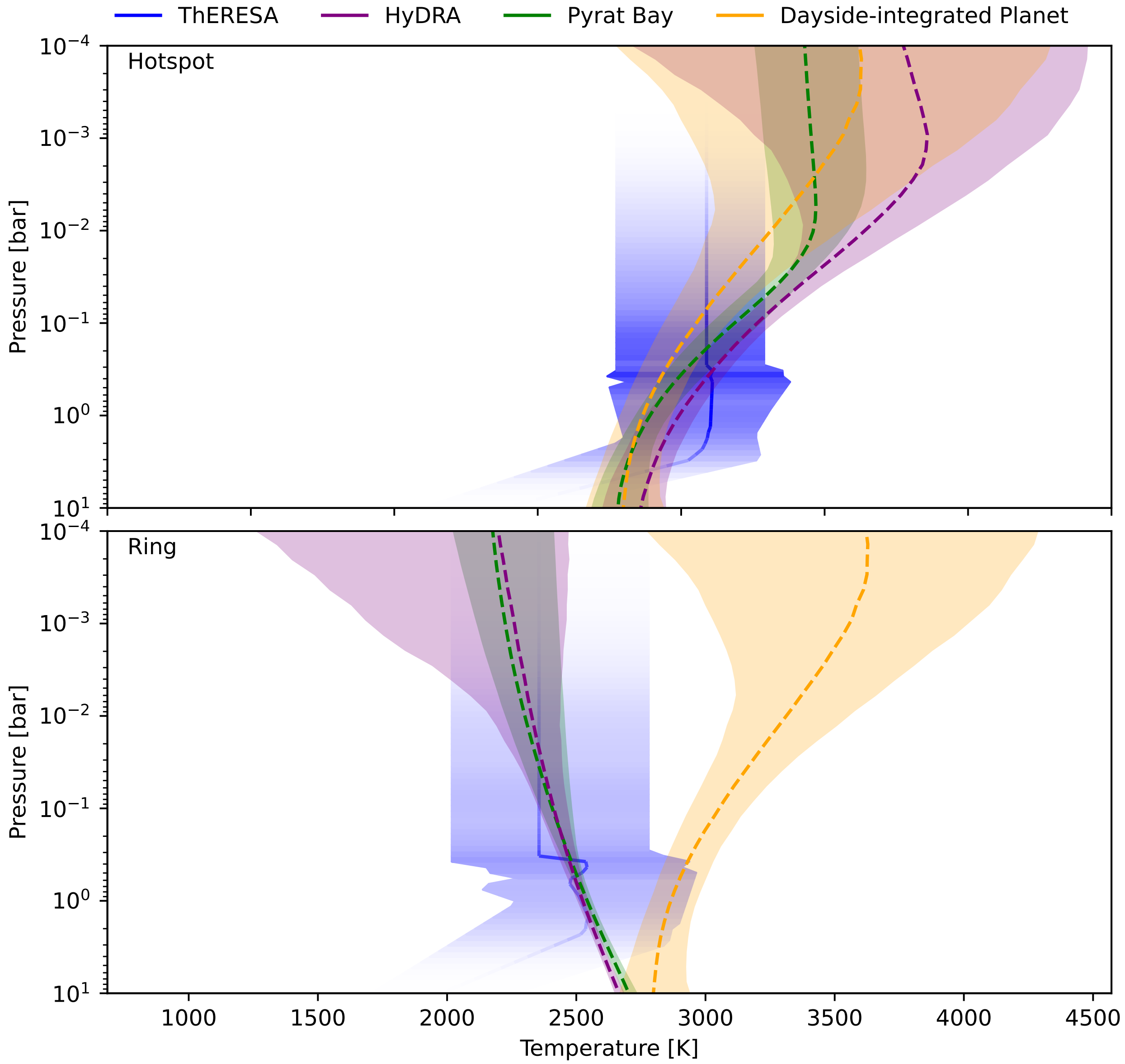

Supplementary Figure 5 | The range of vertical temperature profiles and median (by pressure layer) profile from the best-fitting `ThERESA` 3D temperature map (blue), grouped into the `Eigenspectra` regions and compared against the profiles retrieved from `Eigenspectra` (purple, green) and the full-planet spectrum (orange). The transparency of the range of vertical temperature profiles and the median profile have been scaled by the contribution function at each location, showing which vertical locations are probed by the data. The jagged temperature profiles are caused by the linear interpolation (see text). At the hotspot, in the pressures primarily probed by our observation ($\sim 0.03 - 3$ bar), `ThERESA` finds a mix of inverted thermal profiles near the substellar point (upper bound of the blue region) and more isothermal profiles further from the center of the hotspot. In the ring, the temperature profiles are largely non-inverted at the pressures we probe. In both cases, we see general agreement with the 1D retrievals on the `Eigenspectra`.

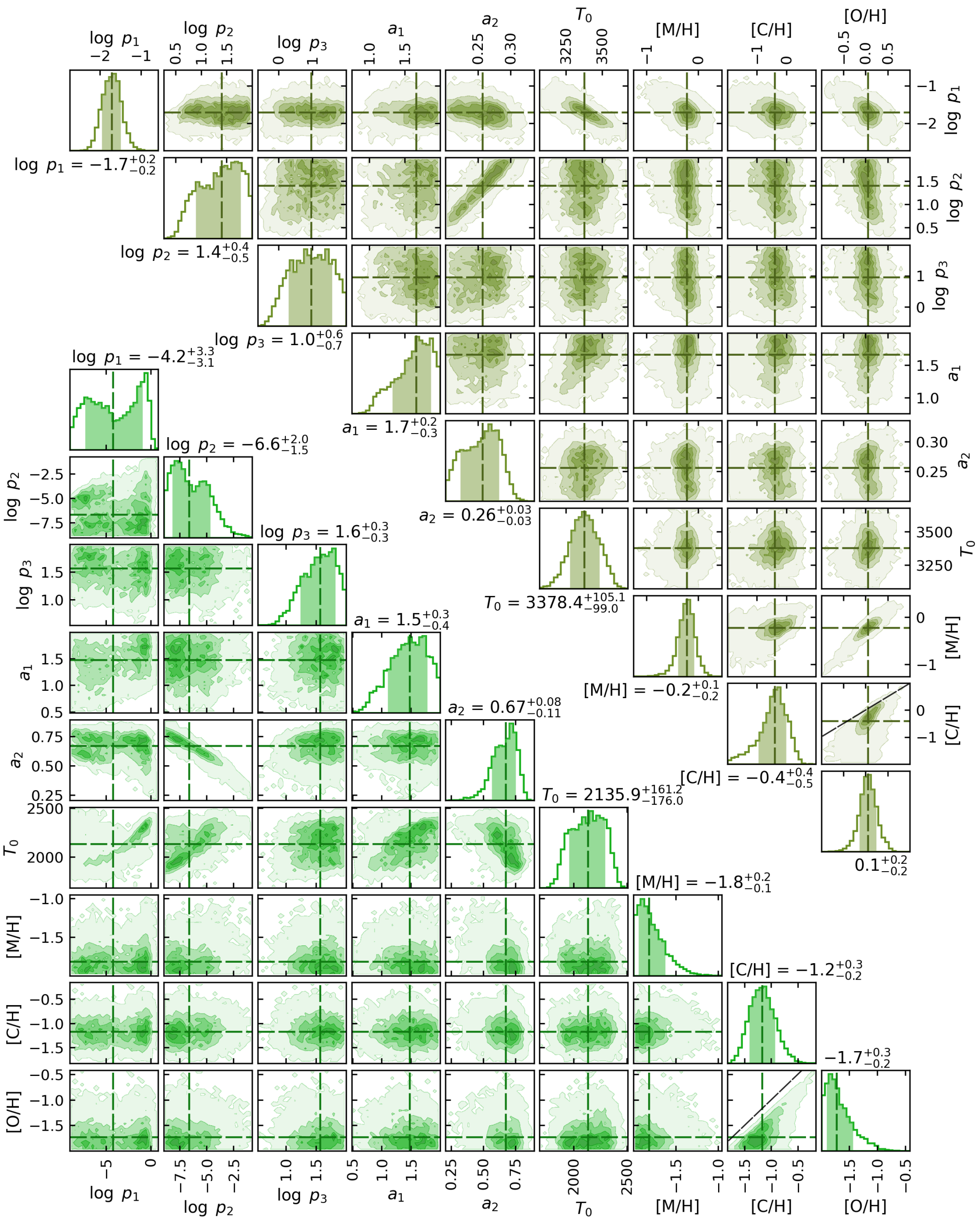

Supplementary Figure 6 | Pairs plot showing posterior distributions for parameters in the `Pyrat Bay` retrieval of the hotspot group (top right) and ring group (bottom left). The first six parameters ($\log p_1$, $\log p_2$, $\log p_3$, a_1, a_2, T_0) determine the P-T profile model[45]. [C/H] and [O/H] are the carbon and oxygen elemental abundances (respectively) relative to solar abundances. [M/H] is a catch-all parameter to scale the abundance of all other metals relative to solar. Off-diagonal plots show 2D posterior probabilities for pairs of parameters, with probability densities shaded in green. On-diagonal plots show marginalized posterior probability distributions for each parameter. Quoted values denote the median and central 68% fraction of the marginal posterior distributions. The black dashed lines trace the constant C/O curve equal to the solar value.

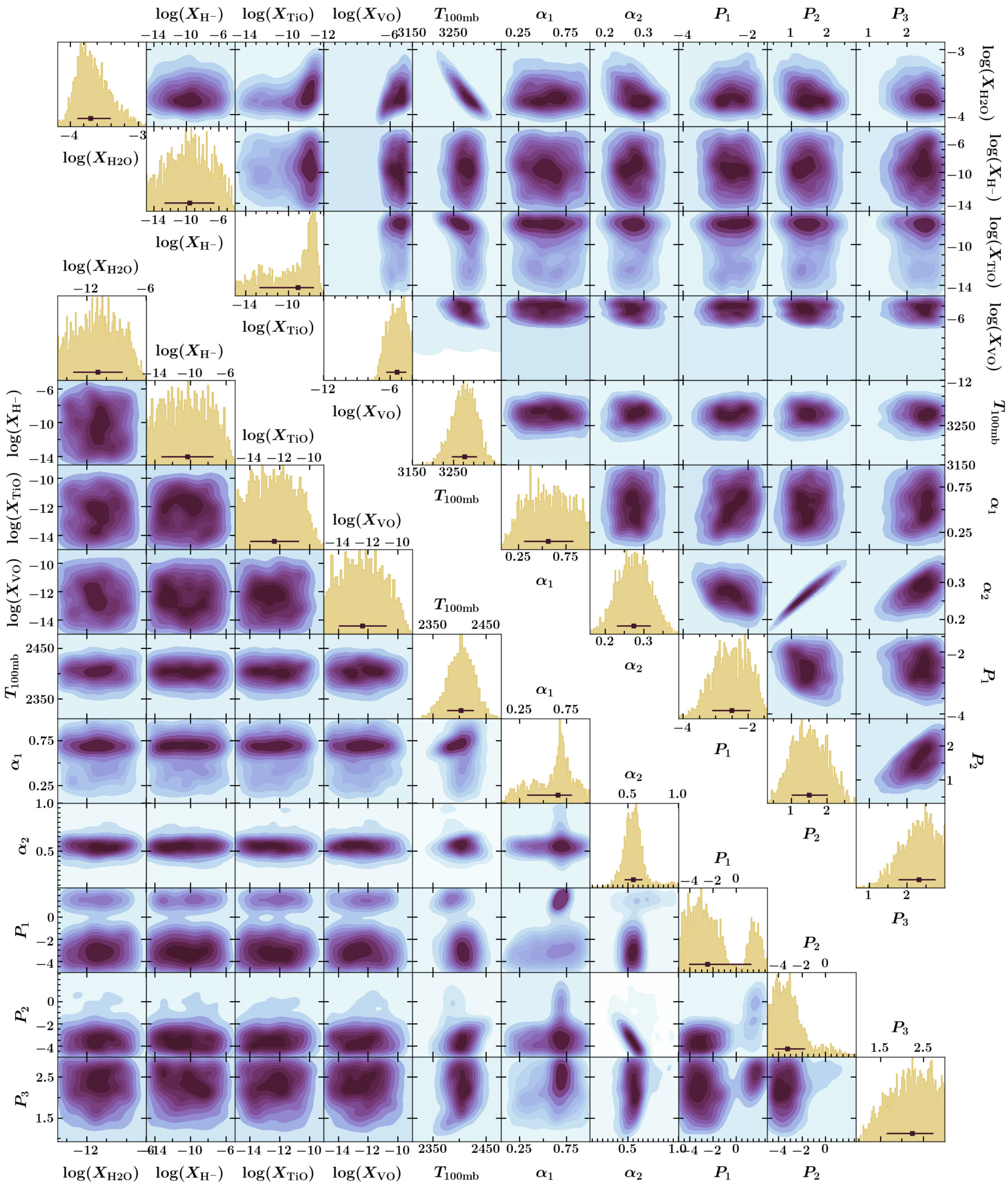

Supplementary Figure 7 | Similar to Supplementary Figure 6, but for the HyDRA retrievals of the hotspot group (upper right) and the ring group standard model (lower left). $\log(X_i)$ are the log mixing ratios of species i, T_{100mb} is the temperature at 100 mbar and α_1, α_2, P_1, P_2 are P-T profile parameters as described in ref[45]. Black points and errors are the median value and 68% credible regions, respectively.

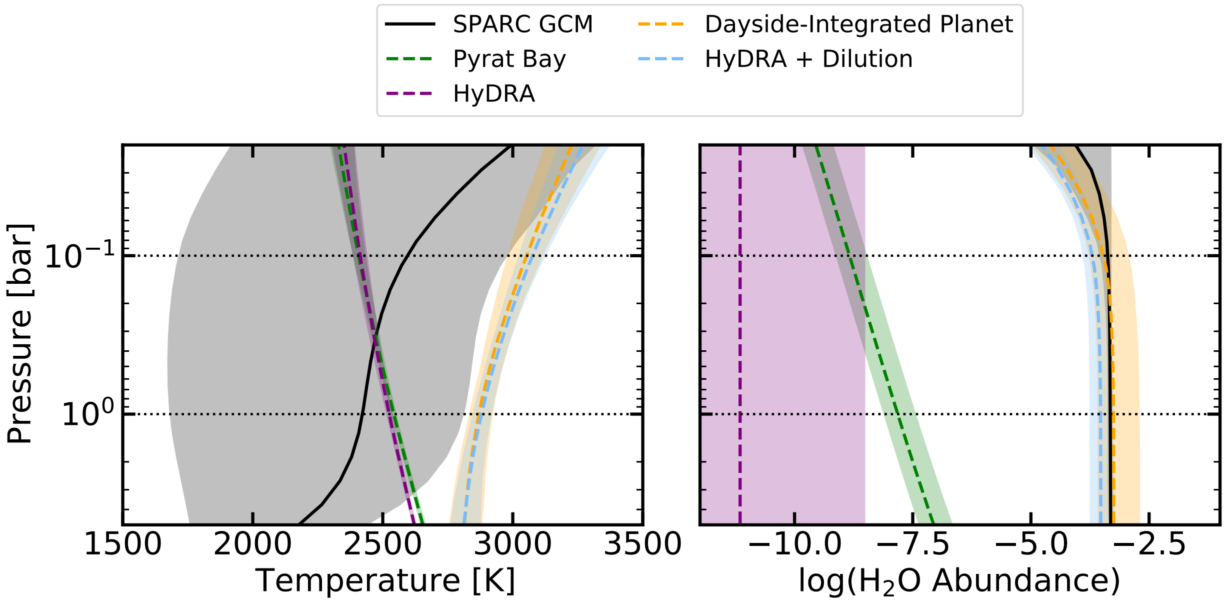

Supplementary Figure 8 | Retrieved T-P profiles (left) and H_2O abundance (right) for the `Eigenspectra` ring group. In all plots, purple and green lines show the standard retrievals following the same set-up as for the hotspot group using `HyDRA` and `Pyrat Bay`, respectively, and yellow lines show the retrieval on the full dayside spectrum[3], with shading showing 1σ confidence intervals. Black solid lines show average profiles in the ring group region from a SPARC/MIT GCM, and black shaded regions show the full range of per-point GCM profiles in that region. We also show one additional retrieval run with `HyDRA` - the standard model with the addition of a dilution parameter (blue lines and 1σ shaded region). The mean posterior value of the dilution parameter is 0.64, with a 2σ credible region of $0.59 - 0.69$. Black dotted lines indicate the approximate extent of pressures probed across all models. Changing the model set-up drastically changes both the T-P profile and the retrieved abundances. We discuss several potential explanations for these changes and future directions for research to better understand these discrepancies in the Methods.

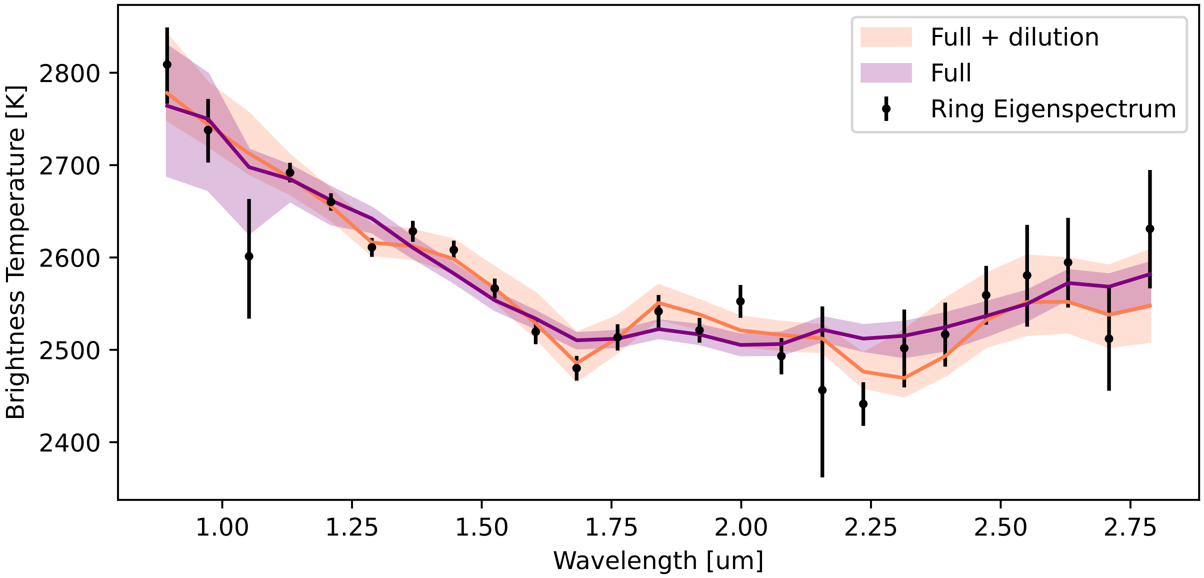

Supplementary Figure 9 | Comparison of resulting spectra from different fits to the `Eigenspectra` ring spectrum (black points with error bars) with `HyDRA`. Purple and orange lines show the standard model and the standard model with the addition of a dilution parameter, respectively. Shaded areas indicate 95.45% credible regions. The standard model with dilution provides the best fit to the data and matches the slight water emission features seen by eye near 1.4 and 1.9 μm, but as discussed in the Methods, the `Eigenspectra` method is designed to eliminate the need for a dilution parameter. Therefore, the preference for the standard+dilution model is likely obscuring some unaccounted-for physical or geometric effects. Error bars are standard deviations from the `Eigenspectra` analysis (see Methods).
